# An exploration of the socio-economic profile of women and costs of receiving abortion services at public health facilities of Madhya Pradesh, India

**DOI:** 10.1186/s12913-017-2159-6

**Published:** 2017-03-21

**Authors:** Sushanta K. Banerjee, Rakesh Kumar, Janardan Warvadekar, Vinoj Manning, Kathryn Louise Andersen

**Affiliations:** 1 Research and Evaluation, Ipas Development Foundation, New Delhi, India; 2 Reproductive & Child Health Programme, Ministry of Health and Family Welfare, Government of India, New Delhi, India; 3 Manager- Research and Evaluation, Ipas Development Foundation, New Delhi, India; 4 Executive Director, Ipas Development Foundation, New Delhi, India; 5 Research and Evaluation, Ipas, Chapel Hill, NC 27516 USA

**Keywords:** Safe abortion, Economic profile of client, Public health facility, Evacuation technology, Cost of abortion

## Abstract

**Background:**

Maternal mortality, which primarily burdens developing countries, reflects the greatest health divide between rich and poor. This is especially pronounced for access to safe abortion services which alone avert 1 of every 10 maternal deaths in India. Primarily due to confidentiality concerns, poor women in India prefer private services which are often offered by untrained providers and may be expensive. In 2006 the state government of Madhya Pradesh (population 73 million) began a concerted effort to ensure access to safe abortion services at public health facilities to both rural and urban poor women. This study aims to understand the socio-economic profile of women seeking abortion services in public health facilities across this state and out of pocket cost accessing abortion services. In particular, we examine the level of access that poor women have to safe abortion services in Madhya Pradesh.

**Methods:**

This study consisted of a cross-sectional client follow-up design. A total of 19 facilities were selected using two-stage random sampling and 1036 women presenting to chosen facilities with abortion and post-abortion complications were interviewed between May and December 2014. A structured data collection tool was developed. A composite wealth index computed using principal component analysis derived weights from consumer durables and asset holding and classified women into three categories, poor, moderate, and rich.

**Results:**

Findings highlight that overall 57% of women who received abortion care at public health facilities were poor, followed by 21% moderate and 22% rich. More poor women sought care at primary level facilities (58%) than secondary level facilities and among women presenting for postabortion complications (67%) than induced abortion. Women reported spending no money to access abortion services as abortion services are free of cost at public facilities. However, poor women spend INR 64 (1 USD) while visiting primary level facilities and INR 256 (USD 4) while visiting urban hospitals, primarily for transportation and food.

**Conclusions:**

Improved availability of safe abortion services at the primary level in Madhya Pradesh has helped meeting the need of safe abortion services among poor, which eventually will help reducing the maternal mortality and morbidity due to unsafe abortion.

## Background

Maternal mortality, which primarily burdens developing countries, reflects the greatest health divide between rich and poor [1]. Although India has made progress towards the Millennium Development Goal of reducing the maternal mortality ratio (MMR) by three quarters, with a 52% reduction between 1990 and the 2007–2009 period, the decline has not been even across all regions of India [2]. Furthermore, abortion related complications continue to account for 8–9% of maternal mortality despite a liberal abortion law [3]. The Medical Pregnancy Termination Act of 1971 allows women to obtain abortion for a range of indications, including contraceptive failure for married women- yet an estimated 50% of abortions are unsafe [4].

Despite strong and favorable policies, India garnered little momentum to improve maternal mortality and morbidity due to unsafe abortion [5], primarily because of limited access to and utilization of safe abortion services. While three-fourths of the Indian population live in rural areas, abortion services are rarely available at rural health facilities because of lack of trained providers [6, 7]. Even where trained providers are available, safe abortion services are underutilized due to numerous individual and community-level factors, such as, lack of awareness of the legality of abortion [8, 9], limited understanding on the implications of unsafe abortion and lack of information on availability of safe providers and methods, poor agency and self-efficacy among women require abortion services, myths, misconception, and social stigma associated with abortion [10, 11].

Reducing maternal deaths requires establishing maternal health services, including safe abortion services by ensuring the presence of qualified staff and increasing use of public sector facilities by women [12–14]. Global studies indicate, however, that a lower percentage of poor and rural women use maternal health services and that cost is an important constraint to service utilization, particularly for the rural poor [2, 12, 13, 15–17]. For abortion care, studies indicate that women prefer services that they believe to be confidential or more affordable, such as private services often offered by untrained or illegal providers [18, 19]. Although private sector abortion care is more expensive, the indirect costs of reaching public sector facilities can be prohibitive to the rural poor community because of long distance travel and repeated visits [14, 20]. This financial burden could be reduced with improved access to women-centered comprehensive abortion care (CAC)^1^ at primary and community health centres located at the rural areas [21].

The state of Madhya Pradesh is among the six states with an MMR higher than the overall India rate (221 compared to 167 deaths per 100,000 live births) [2]. Madhya Pradesh is one of India’s largest states with about three fourths of its population living in rural areas [22]. Previous studies have documented low use of maternal health facilities in Madhya Pradesh, especially in rural areas [13]. Furthermore, studies have shown that use of unsafe abortion methods in Madhya Pradesh is common, resulting in high levels of post-abortion complications. In 2006, when only 2.7% of PHCs offered abortion services, the state government of Madhya Pradesh with technical support of Ipas Development Foundation (IDF) began a concerted effort to ensure access to safe abortion services at all levels of public health facilities [23], including primary, secondary facilities, and tertiary facilities. Key IDF interventions involved training physicians, orienting nursing staff, providing essential equipment and drugs, and establishing site signage (poster or wall sign) on availability of abortion services [24]. The primary goal of this effort was to ensure free access to safe and early abortion services among rural and urban poor.

This study explores the socio-economic profile of women accessing safe abortion services at different levels of public health facilities in rural and urban Madhya Pradesh. A recent study by Chaturvedi et al. examined availability of abortion services at public and private facilities across three provinces of Madhya Pradesh. Findings showed low availability of safe abortion among public health facilities, particularly in rural areas. While the study provided critical information on the capacity for and quality of abortion care at the facility level, the question still remains as to the safe abortion practices of the poorest women in Madhya Pradesh [25]. This study specifically examines: 1) whether poor women were accessing safe abortion services at public health facilities, 2) the socio-economic profile of women varied by type of public sector facilities, and 3) if women incurred any out-of-pocket cost (indirect) to access services in terms of transportation, food, clinical examination, and medicines.

While research has explored the use of maternal health and abortion services in tertiary and secondary level facilities, there has been no examination of use of abortion services across all levels of the public health system in India [26]. Having a better understanding of who is using the free abortion services at public health facilities is crucial to developing appropriate strategies to reach all women.

## Methods

### Design and site selection

**Fig. 1:**
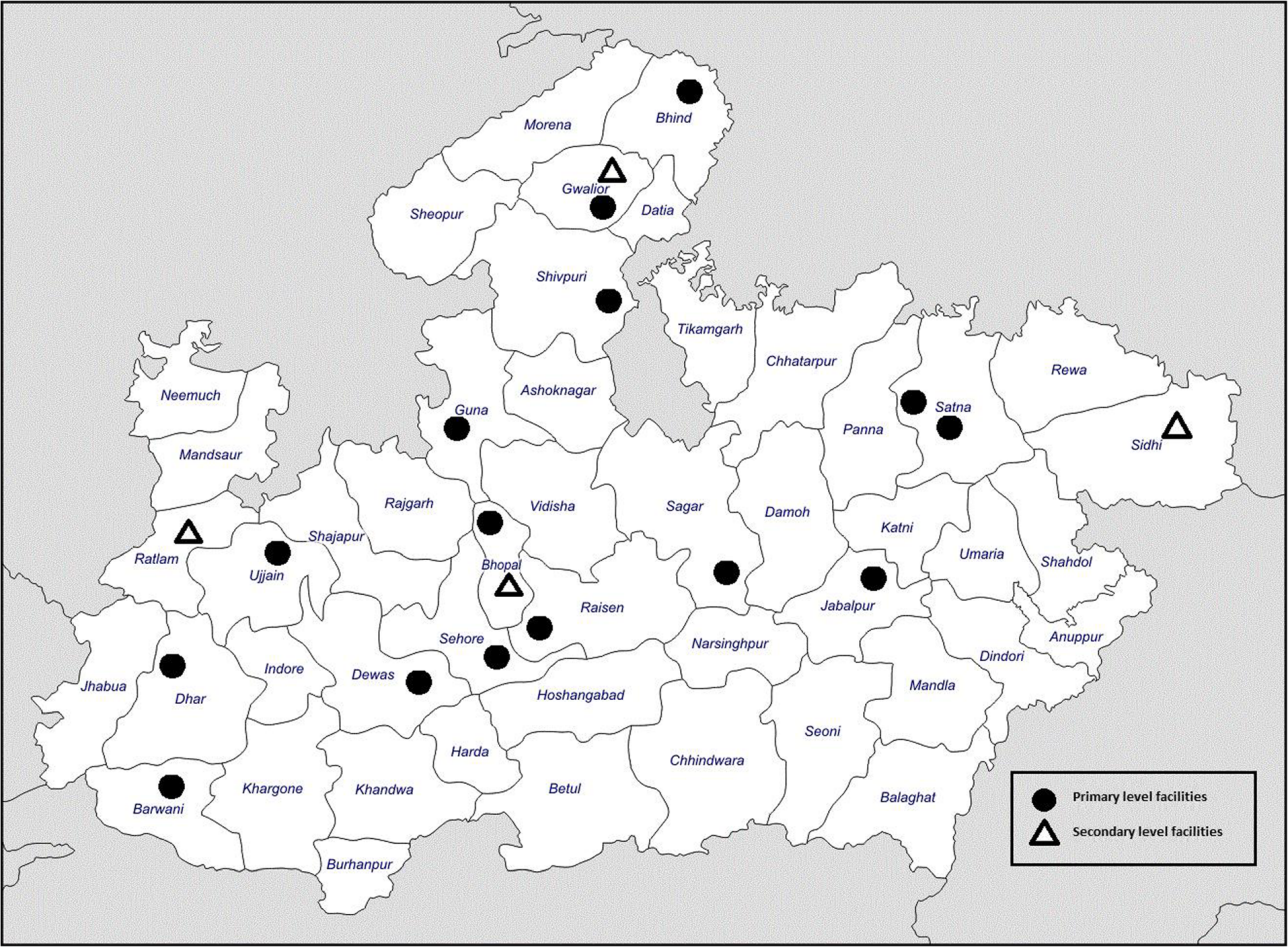
Location of 19 sampled public sector facilities, Madhya Pradesh, India 2014. Figure was created by the authors

This study employed a cross-sectional design. The protocol for this study was reviewed and approved by the state government of Madhya Pradesh, IRB boards at the Center for Media Studies in India. A total of 20 public health facilities in Madhya Pradesh were selected using a two-stage sample design. First, all facilities offering CAC in the 50 districts of Madhya Pradesh were categorized according to the four regions based on government designations of health divisions (12–13 districts per division). Facilities were also stratified by type (primary vs secondary) within each region. Secondly, four primary level facilities and one secondary level hospital were selected from each region using computer generated random numbers. A total of 20 health facilities were identified for the study across 16 districts of the Madhya Pradesh. However, one health facility dropped out at the beginning as they stopped providing abortion services. The 19 public health facilities selected for inclusion are shown in Fig. 1.

### Sample size

The sample size was estimated for the percentage of poor women accessing abortion services from public health facilities. For operational reasons, poor women were defined as those women holding a below poverty line (BPL) card issued by the state government. The BPL designation is a composite index score of household economic status defined by 13 socio-economic indicators, including landholding, type of house, availability of clothes, food security, sanitation, ownership of consumer durables, literacy status, status of household labour force, means of livelihood, status of school-going children, type of indebtedness, reason of migration and preference of assistance [27]. We estimated that primary health centres, consisting of one trained medical doctor, would have the lowest caseloads of abortion clients followed by community health centres, sub-district, and district hospitals. A primary health centre (PHC) is the first contact between the rural community and a medical doctor and cover around 30,00 population; community health centres (CHCs) are referral centres for PHCs and cover around 120,000 rural population. Secondary level facilities including, sub-district (SDH) and district hospitals (DH) are located at towns and cities and provide specialized health care services to both rural and urban population [28].

On the basis of available research in the state [29], we estimated that women from BPL category would be approximately 20 to 25% at different levels of public health facilities (20% at PHC, 25% at CHC, and 25% at SDH/DH hospitals). The following formula was used to estimate the minimum sample size:

$$ \mathrm{q}/\mathrm{n}\mathrm{p}=\mathrm{c}{\mathrm{v}}^2 $$

Where the coefficient of variation (cv) is fixed at 0.1 or 10% (equivalent to fixing absolute error at 20% of true proportion and estimating at 95% confidence) and p equals the proportion of women holding BPL card. This suggested a total minimum sample size of 1000 women accessing abortion services at randomly selected public health facilities.

### Study participants, tools, and data management

Women presenting to selected facilities with abortion and post-abortion complications and provided informed consent were interviewed between May and December 2014. Two separate data collection tools were used: a structured case logbook and a structured questionnaire to capture individual and household economic status.

As a routine protocol of the CAC intervention, the logbook was used for all women seeking abortion-related services, including induced abortion and postabortion complications. Logbooks were maintained by the CAC trained medical doctor or nursing staff and contain individual-level data on abortion services, including age, gestational age of pregnancy, diagnosis (induced or incomplete abortion), uterine evacuation technology, reasons for pregnancy termination, provider identification, procedure date, and postabortion contraceptive uptake/method. To ensure privacy and confidentiality, duplicate pages of abortion log-books were collected for the study, which excluded women’s identifying information (name, address and name of guardian, where applicable).

The individual and household level data collection tool was developed to provide a comprehensive client profile. Questions included whether the client belonged to one of the castes associated with the most disadvantage groups scheduled castes (SC), scheduled tribes (ST) and other backward caste (OBC), if the family held a BPL card, as well as education and occupation of abortion clients and their spouses. To assess household economic status, a series of questions were asked on the possession of household durables such as appliances and means of transportation. In addition, clients were asked about the type of house they lived in, whether it was owned or rented, in-home access to separate toilet, electricity, cooking fuel, and land holding and quality of land holding in terms of irrigation facility. Finally, women described indirect out-of-pocket costs incurred for transportation, medicine and clinical tests, and food.

Participants were interviewed by the CAC trained doctor who provided the abortion service or nursing staff responsible for documentation, instrument processing and post-abortion counselling. One doctor and one nursing staff from each of the primary level facilities were trained on the interview process, collection of consent form and data collection techniques. At facilities with higher caseloads, one additional nursing staff was trained to ensure that at least one trained person always remained available at the study facility. Providers recorded service data in the facility logbooks; IDF staff visited each facility at least once every two months to collect carbon copies that exclude patient identifiers. All women who received abortion related services were approached for the interview. The study staff explained the purpose and nature of the study to all participants and assured women of low literacy levels that they could contact the study staff at any time to reread or to explain the informed consent form to them. Women were primarily interviewed by the trained nursing staff at the post-abortion recovery room where no other patient or staff were available during interviews. All participants signed the informed consent form or gave their verbal consent to the interviewer who noted this consent on the paper form. Participant confidentiality was ensured by using a client identification number on data collection forms. No compensation was provided to participants, and they were assured that study non-participation would in no way affect their service provision and care.

### Analysis

Data were entered and analysed using SPSS version 13.0 (IBM, Armonk, NY, USA). Descriptive statistics were computed, including frequency and percent of non-missing for categorical variables and means with associated standard deviations for continuous variables.

Past research in India has relied on caste and holding a BPL card as proxies for poverty [14, 30], however these measures are not sensitive enough to measure differences among poor populations. Alternatively, a number of studies have demonstrated that the wealth index is a good proxy of economic status [31–34] and has been used extensively to explain the economic differentials in demographic and health parameters of developing countries. For the purpose of this study, we have adapted the DHS Wealth Index [31]. In addition, we have validated the wealth index and examined its linkages with individual proxy variables of social and economic status, which includes education, caste, per-capita household income, land-holding, occupation, and living standard.

To develop the wealth index for this study, we first used principal component analysis (PCA) where each household asset (as reported by the study respondent) was assigned a weight (factor score) generated through PCA analysis. The resulting asset scores were standardized based on a normal distribution. Next, each individual household was then assigned a score for each of the 27 selected economic variables, and a composite wealth index score was computed by adding individual items. Finally, the composite wealth index scores were segmented into three categories and defined as poor, middle and rich. Reliability and internal consistency of the wealth index were examined through α (alpha) score, while the validity of the wealth index was assessed by cross classifying the wealth index with reported education, caste, landholding, household income and BPL card holding. The distribution of the composite wealth index was normally distributed.

Because public sector facilities do not charge any direct fees for providing abortion services, indirect costs were analysed for transportation, medicine, food and clinical tests and summed to calculate total and average out-of-pocket costs incurred by each respondent for accessing abortion services. Where appropriate, a currency conversion rate of 1 USD: 64 INR was used (Reference date June 2015).

All analyses comparing service use, abortion experience and economic expenditure were carried out separately for the primary (including PHCs and CHCs) and secondary (district and sub-district hospitals) level health facilities and facility level variations were examined using statistical significance tests.

## Results

A total of 1036 women presenting to study facilities for abortion and post-abortion complications and provided informed consent were interviewed between May and December 2014. Seventy-two percent (*n* = 742) of respondents were interviewed at selected primary level health facilities (primary and community health centres) and 28% were interviewed at secondary level hospitals (including sub-district and district hospitals). Although no women refused to participate, seven interviews remained incomplete because women had to leave to catch their scheduled public transport.

### Characteristics of study participants

Among the 1036 study participants who received abortion services at public health facilities, most (75%) were 20–29 years of age (Table 1). Nearly three-fourths (74%) of women identified themselves as belonging to a scheduled caste/tribe or other backward class.^2^ Almost three-fourths of women (76%) lived in rural areas and 38% of respondents reported no or primary level of education. More than half of women (60%) didn’t work outside their homes in the past year. The main source of household income among women interviewed at primary level facilities was from owning a small farm (50%) or from a daily wage (18%), while women at secondary level hospitals reported salary (33%) and business (21%) as the main source of income. Ownership of BPL (below poverty line) card was similar among women visited primary (37%) and secondary (35%) level health facilities.

**Table 1:** Socio-demographic profile of study respondents received abortion services at selected primary and secondary level public health facilities, Madhya Pradesh, India 2014

	Primary facilities (*n* = 742)		Secondary facilities (*n* = 294)		*p*-value	All facilities (*n*=1036)	
	*n*	%	*n*	%		*n*	%
Age							
15–19	33	4.4%	8	2.7%	0.199	41	4.0%
20–24	294	39.6%	89	30.3%	0.005	383	37.0%
25–29	282	38.0%	108	36.7%	0.703	390	37.6%
30–34	101	13.6%	56	19.0%	0.027	157	15.2%
35 & above	32	4.3%	33	11.2%	0.000	65	6.3%
Mean age (SD)	25 (4.4)		27 (5.16)		0.000	26 (4.65)	
Caste							
Scheduled caste (SC)	112	15.1%	33	11.2%	0.105	145	14.0%
Scheduled tribe (ST)	161	21.7%	26	8.8%	0.000	187	18.1%
Other backward caste (OBC)	308	41.5%	122	41.5%	0.996	430	41.5%
General	161	21.7%	113	38.4%	0.000	274	26.4%
Place of residence							
Urban	153	20.6%	95	32.3%	0.000	248	23.9%
Rural	589	79.4%	199	67.7%	0.000	788	76.1%
Education of respondent (max number of year)							
Non-literate or Primary (4)	311	41.9%	83	28.2%	0.000	394	38.0%
Middle standard (9)	198	26.7%	68	23.1%	0.237	266	25.7%
Secondary (11)	140	18.9%	63	21.4%	0.349	203	19.6%
Higher secondary & above (15)	93	12.5%	80	27.2%	0.000	173	16.7%
Education of spouse							
Non-literate or Primary (4)	186	25.1%	43	14.6%	0.000	229	22.1%
Middle standard (9)	155	20.9%	55	18.7%	0.431	210	20.3%
Secondary (11)	207	27.9%	69	23.5%	0.146	276	26.6%
Higher secondary (12)	96	12.9%	63	21.4%	0.000	159	15.3%
Graduate & above (17)	98	13.2%	64	21.8%	0.000	162	15.6%
Occupation of respondent							
Cultivation/ agricultural labour	222	29.9%	34	11.6%	0.000	256	24.7%
Non-agricultural labour	72	9.7%	21	7.1%	0.071	93	9.0%
Salaried	36	4.9%	16	5.4%	0.054	52	5.0%
Business and other	17	2.3%			0.000	17	1.6%
Not working	395	53.2%	223	75.9%	0.759	618	59.7%
Occupation of spouse							
Cultivation/ agricultural labour	374	50.4%	67	22.8%	0.228	441	42.6%
Non-agricultural labour	137	18.5%	62	21.1%	0.333	199	19.2%
Salaried	108	14.6%	96	32.7%	0.000	204	19.7%
Business and other	83	11.2%	61	20.7%	0.000	144	13.9%
Not working	40	5.4%	8	2.7%	0.065	48	4.6%
Owning a BPL card	272	36.7%	102	34.7%	0.553	374	36.1%
Per capita household income [INR] (sd)	1354 (1626)		1910 (2241)		0.000	1513 (1839)	

### Characteristics of abortion services

Women travelled an average distance of 13 km to reach a primary level facility and 26 km to reach to a secondary level hospital. More than two-thirds (70%) of study respondents received induced abortion services, while the remaining 30% received services for post-abortion complications, including incomplete abortion. However, the proportion of women seeking care for post-abortion complications was significantly higher at secondary level hospitals (48%) compared to primary level facilities (23%). Nearly all women (97%) requested abortion services during the first trimester (within 12 weeks of gestation). Almost 95% women received evacuation through WHO/GOI recommended technology, including MVA (51%), MA (33%) and EVA (11%). Almost 90% women received a modern contraceptive method immediately after the abortion procedure (Table 2).

**Table 2:** Characteristics of abortion services received by study respondents at selected primary and secondary level public health facilities, Madhya Pradesh, India 2014

	Primary facilities (*n* = 742)		Secondary facilities (*n* = 294)		*p*-value	All facilities (1036)	
	*n*	%	*n*	%		*n*	%
Diagnosis							
Induced	575	77.5%	154	52.4%	0.000	729	70.4%
Incomplete abortion	167	22.5%	140	47.6%	0.000	307	29.6%
Gestation (duration of pregnancy)							
6–8 weeks	661	89.1%	256	87.1%	0.360	917	88.5%
8–12 weeks	66	8.9%	22	7.5%	0.462	88	8.5%
More than 12 weeks	15	2.0%	16	5.4%	0.004	31	3.0%
Mean gestation (SD)		7.0 (1.9)		7.5 (2.6)	0.002		7.1 (2.2)
Abortion methods							
MVA	380	51.2%	150	51.0%	0.955	530	51.2%
EVA	61	8.2%	48	16.3%	0.000	109	10.5%
MA	292	39.4%	53	18.0%	0.000	345	33.3%
D&C	9	1.2%	43	14.6%	0.000	52	5.0%
Mean distance travelled in km (sd)	12.6 (35.3)		25.7 (26.38)		0.000	16.3 (33.54)	
Received post-abortion contraceptives	692	93.3%	240	81.6%	0.000	932	90.0%
Reference source (referred by)							
Outreach workers (ANM,ASHA,AWW)	172	23.2%	44	15.0%	0.003	216	20.8%
NGO workers	2	0.3%	1	0.3%	0.848	3	0.3%
Others	568	76.5%	249	84.7%	0.003	817	78.9%
Accompanied to the facility by outreach workers	134	18.1%	40	13.6%	0.083	174	16.8%

### Economic profile of women accessed abortion services at public health facilities

Classification of study respondents based on their individual wealth score suggests that women who accessed abortion services at primary level health facility around 58% of them belonged to poor households followed by 22% and 20% from middle and rich households, respectively (Table 3). Among women who visited secondary level urban hospitals 52, 20 and 28% belonged to poor, middle, and rich households, respectively.

**Table 3:** Economic profile of study respondents received abortion services at selected primary and secondary level public health facilities, Madhya Pradesh, India 2014

Economic profile	Primary facilities (*n*=742)		Secondary facilities (*n*=294)		*p*-value	All facilities (*n*=1036)	
	*n*	%	*n*	%		*n*	%
Poor	432	58.2%	153	52.0%	0.070	585	56.5%
Middle	163	22.0%	58	19.7%	0.427	221	21.3%
Rich	147	19.8%	83	28.2%	0.000	230	22.2%

### Characteristics of service provision by economic profile of women

Among women who visited primary level facilities for induced abortion services, 55% were poor, 23% middle income and 22% rich, while women who presented for incomplete abortion were 68% poor, 19% middle income, and 13% rich (Table 4). A similar trend was observed at secondary level hospitals. However, women who visited secondary level facilities for induced abortion were relatively well off than the women visited primary level facilities (Primary 22% Vs Secondary 38%, *p* < 0.001).

**Table 4:** Presenting induced and incomplete abortion by economic profile of women, Madhya Pradesh, India 2014

Economic Status	Primary facilities (*n*=742)				Secondary facilities (*n*=294)						All facilities (*n*=1036)			
	Induced (*n*=575)		Incomplete (*n*=167)		Induced (*n*=154)			Incomplete (*n*=140)			Induced (*n*=729)		Incomplete (*n*=307)	
	*n*	%	*n*	%	*n*	%	*p*-value	*n*	%	*p*-value	*n*	%	*n*	%
Poor	319	55.5%	113	67.7%	60	39.0%	0.000	93	66.4%	0.664	379	52.0%	206	67.1%
Moderate	131	22.8%	32	19.2%	36	23.4%	0.876	22	15.7%	0.157	167	22.9%	54	17.6%
Rich	125	21.7%	22	13.2%	58	37.7%	0.000	25	17.9%	0.179	183	25.1%	47	15.3%

There was no statistically significant association between economic profile and uterine evacuation method, although relatively more rich women appeared to receive medical abortion as compared to poor and middle income women (Table 5).

**Table 5:** Uterine evacuation technology used at public health facilities by economic profile of women, Madhya Pradesh, 2014

Economic Status	Primary facilities (*n*=742)				Secondary facilities (*n*=294)						All facilities (*n*=1036)			
	Surgical (*n*=441)		Medical (*n*-301)		Surgical (*n*=198)			Medical (*n*=96)			Surgical (*n*=639)		Medical (*n*=397)	
	*n*	%	*n*	%	*n*	%	*p*-value	*n*	%	*p*-value	*n*	%	*n*	%
Poor	265	60.1%	167	55.5%	106	53.6%	0.120	47	49.0%	0.260	371	58.1%	214	53.9%
Moderate	101	22.9%	62	20.6%	40	20.2%	0.446	18	18.8%	0.694	141	22.1%	80	20.2%
Rich	75	17.0%	72	23.9%	52	26.3%	0.006	31	32.3%	0.103	127	19.9%	103	25.9%

### Indirect cost of accessing abortion services at public health facilities

Although abortion services are technically free at public sector facilities, women reported that the indirect average total cost for an abortion at primary health centre was 67 INR (1USD) (SD = 137 INR; Range: 0–2700), while the same cost was almost three times for women accessed services at urban hospitals (Table 6).

**Table 6:** Indirect cost (in INR) of accessing abortion services as reported by study respondents received abortion services at selected primary and secondary level public health facilities, Madhya Pradesh, India 2014

	Primary facilities		Secondary facilities		*p*-value	All facilities	
	Mean (SD)	Range	Mean (SD)	Range		Mean (SD)	Range
Average cost	67 (137.31)	(0–2700)	239 (357.87)	(0–3000)	0.000	116 (236.19)	(0–3000)
Cost by components							
Transportation	42 (105.75)	(0–2400)	181 (297.81)	(0–3000)	0.000	81 (192.44)	(0–3000)
Medicines	8 (32.0)	(0–130)	7 (41.46)	(0–400)	0.681	8 (34.93)	(0–400)
Clinical tests	--	(0–300)	40 (124.68)	(0–950)	NE	12 (69.37)	(0–950)
Local stay	2 (20.26)	(0–300)	2 (14.15)	(0–200)	NE	2 (18.72)	(0–300)
Other costs	14 (51.99)	0–900)	10 (39.10)	(0–300)	0.245	13 (48.70)	(0–900)
Cost by wealth Status							
Poor	69 (105.85)	(0–1000)	305 (425.15)	(0–3000)	0.000	131 (257.09)	(0–3000)
Middle	56 (76.88)	(0–320)	188 (266.12)	(0–1610)	0.000	91 (161.61)	(0–1610)
Rich	75 (236.31)	(0–2700)	154 (236.84)	(0–3000)	0.023	103 (239.06)	(0–2700)

These expenses were primarily associated with travel costs, and varied from 67 INR (1 USD) at primary level facilities to 239 INR (3 USD) at urban hospitals with a large range (0–3000 INR). Other expenses reported by a few women included medicines and clinical tests. However, expenses for clinical tests were only mentioned by women who received care at urban hospitals. The total indirect cost of accessing abortion services had no significant association with women’s economic profile at primary level facilities. In contrast, significant associations can be observed at the secondary level facilities. Surprisingly, poor women spent more on average (305 INR) than their counterparts from middle (188 INR) and rich households (154 INR), when seeking care at urban hospitals. In general, indirect cost of accessing abortion services were more at the secondary level facilities. Among poor women who visited secondary level urban hospitals spent almost five times more than poor women who visited primary level facilities (305 INR vs 69 INR; *p* < 0.001), while women from middle (56 INR vs 188 INR; *p* < 0.001), and rich (75 INR vs 154 INR; *p* < 0.023), households spent three and two times more respectively.

## Discussion

This is one of the first studies in India, following the decentralization initiative begun in 2006, that both examines the profile of women seeking safe abortion services at public sector facilities and assesses this access in the context of poverty. Findings from our representative sample of public health facilities suggest that women coming for abortion services are predominantly from rural areas; even at urban hospitals, more than two-thirds of women came from rural areas. The majority of women who visited public health facilities to access abortion services are poor, who are at highest risk of maternal mortality and morbidity due to unsafe abortion. This finding should be understood in the context of health inequity in India, where health service utilization has been found to differ significantly by caste and income. Results from a study looking at progress based on social strata showed extreme inequality in utilization of health services by caste. For example, in 2005–06, immunization coverage among scheduled tribes and scheduled castes was 31.3 and 39.7% respectively, compared with 53.8% among other castes, and absolute inequalities between these castes increased with time [20]. There are several reasons why the cost of abortion may be higher among poor women. First, poor women mostly came from the rural area and had relatively higher cost of travel and food than those in towns and villages closer to facilities. Moreover, poor women, perhaps owing to their delayed treatment, also visited a higher level facility to treat their incomplete abortion; in these facilities, women were more likely to be asked for clinical examination and pay higher fees.

To validate these findings and further to understand the relative profile of women categorized as ‘poor’, ‘middle’ and ‘rich’, we assessed the linkages of these three economic segments with the individual household economic profile. The analysis suggests that the majority of women grouped as poor women came from rural areas and self-reported low literacy, disadvantaged caste, and living in a *kachha* house without a toilet or modern cooking fuel. Contrary to our expectations, the association with economic profile and holding a BPL card was not strong. Only 44% of poor women reported holding a BPL card, while almost 17% of rich women reported owing the BPL card. However this finding is in line with an earlier study that found that BPL card distribution has failed to reach the majority of the poor households in India, while a portion end up with users from non-poor households [27]. With the exception of BPL, all other socio-economic variables validate our classification of women into poor, middle and rich households, supporting the validity of our findings that decentralization of abortion services is resulting in access to services among women of all economic profiles, and predominantly the poor.

This study suggests that public sector facilities provide access to postabortion services for all women, especially poor and vulnerable populations because the majority of women seeking care met our classification for poor. Although poor women do use public health facilities, the fact that they are the predominant users of *post-abortion* care suggests a lack of access to information and services related to safe abortion and a reliance on informal providers for initial pregnancy termination. This finding is in line with other studies on utilization of maternal health services that suggest that poor women have lower rates of formal health care utilization overall. Results from an analysis of three rounds of National Family Health Survey found that while use of antenatal care (ANC) services in the whole of India increased by 12 percentage points between 1992 and 2006, the increase among the poor was only 0.1 percentage points and that use of skilled birth attendants had increased by 13 percentage points, while only 2 percentage points could be attributed to women belonging to the poorest quintile [14]. Another study similarly found significant improvement in institutional delivery for the non-poor, with women in the richest quintile being six times more likely to deliver in an institution than were those in the poorest quintile [20].

Cost of abortion has often been regarded as one of the major barriers to accessing safe abortion services. Studies indicate that availability of free services may not ensure service utilization due to the burden of additional out-of-pocket expenses [20]. Abortion services are technically free at public health facilities; however, women in this study reported spending 67 INR (1 USD) at primary health facilities and 239 INR (4 USD) at secondary level hospitals primarily because of transportation, as well as other expenses including food and clinical tests. In addition, the average indirect expenditures varied significantly depending on distance travelled and mode of transportation. Surprisingly poor women who visited urban hospitals spent almost thrice that of rich women (305 INR compared to 156 INR), while no difference in costs between poor and rich women was observed for rural, primary level facilities. Thus the decentralization of abortion services to the periphery not only facilitates access to safe abortion services for poor women, but also reduces the financial burden due to long distance travel and loss of working days. It should be noted that the indirect out-of-pocket costs reported here are significantly lower than costs reported in other studies [35, 36].

The findings of this study must be viewed in light of methodological limitations. Results cannot be generalized to the whole of Madhya Pradesh as this study was restricted to the public sector facilities. Economic status of the households was determined using self-reported assets and access to consumer durables and as such are susceptible to bias. Furthermore, the cross-sectional nature of the study does not allow us to accurately measure the impact of decentralization of abortion services on poor women’s access to care.

## Conclusions

Although poor women are predominantly visiting public health facility, many poor still do not have access to correct information and services which lead them to unsafe providers with aggravated risk of postabortion complications. Safe accessible and affordable abortion services should continue to be scaled and supported across public facilities in Madhya Pradesh, and India, particularly in disadvantaged areas of India.

## Abbreviations

BPL: Below poverty line
CAC: Comprehensive abortion care
CHC: Community Health Centre
D&C: Dilatation and curettage
DH: District Hospital
EVA: Electric vacuum aspiration
INR: Indian rupee
KM: Kilo meter
LPG: Liquid petroleum gas
MA: Medical abortion
MCH: Medical College Hospital
MDG: Millennium development goal
MVA: Manual vacuum aspiration
PCA: Principal component analysis
PHC: Primary Health Centre
SDH: Sub-district hospital

## Footnotes

1 Ipas’s woman-centered CAC approach takes into account a woman’s physical and mental health needs as well as her personal circumstances and her ability to access services [21]. CAC should consist of high-quality abortion, postabortion and family planning services that are decentralized, affordable, sustainable and tailored to the needs of all women, including youth. The aim of CAC services are to reduce unintended pregnancies and abortion [21]].

2 Under Article 340–342 of the Indian Constitution, the Government of India classifies some of its citizens based on their social and economic conditions as Scheduled Caste (SC), Scheduled Tribes (ST), and Other Backward Class (OBC). The term backward class is a collective term, used by the government for castes that are economically and socially disadvantaged.
